# COVID-19 and Individual Genetic Susceptibility/Receptivity: Role of ACE1/ACE2 Genes, Immunity, Inflammation and Coagulation. Might the Double X-Chromosome in Females Be Protective against SARS-CoV-2 Compared to the Single X-Chromosome in Males?

**DOI:** 10.3390/ijms21103474

**Published:** 2020-05-14

**Authors:** Donato Gemmati, Barbara Bramanti, Maria Luisa Serino, Paola Secchiero, Giorgio Zauli, Veronica Tisato

**Affiliations:** 1 Department of Morphology, Surgery and Experimental Medicine and Centre Haemostasis & Thrombosis, University of Ferrara, 44121 Ferrara, Italy; 2 University Centre for Studies on Gender Medicine, University of Ferrara, 44121 Ferrara, Italy; barbara.bramanti@unife.it; 3 Department of Biomedical & Specialty Surgical Sciences, University of Ferrara, 44121 Ferrara, Italy; 4 Department of Medical Sciences and Centre Haemostasis & Thrombosis, University of Ferrara, 44121 Ferrara, Italy; maria.luisa.serino@unife.it; 5 Department of Morphology, Surgery and Experimental Medicine and LTTA Centre, University of Ferrara, 44121 Ferrara, Italy; paola.secchiero@unife.it (P.S.); giorgio.zauli@unife.it (G.Z.); veronica.tisato@unife.it (V.T.)

**Keywords:** SARS-CoV-2, COVID-19, *ACE1*, *ACE2*, RAS-pathway, *TMPRSS2*, sex/gender-gap, lung shut-down, prognostic molecular/genetic markers, inflammation, thrombosis

## Abstract

In December 2019, a novel severe acute respiratory syndrome (SARS) from a new coronavirus (SARS-CoV-2) was recognized in the city of Wuhan, China. Rapidly, it became an epidemic in China and has now spread throughout the world reaching pandemic proportions. High mortality rates characterize SARS-CoV-2 disease (COVID-19), which mainly affects the elderly, causing unrestrained cytokines-storm and subsequent pulmonary shutdown, also suspected micro thromboembolism events. At the present time, no specific and dedicated treatments, nor approved vaccines, are available, though very promising data come from the use of anti-inflammatory, anti-malaria, and anti-coagulant drugs. In addition, it seems that males are more susceptible to SARS-CoV-2 than females, with males 65% more likely to die from the infection than females. Data from the World Health Organization (WHO) and Chinese scientists show that of all cases about 1.7% of women who contract the virus will die compared with 2.8% of men, and data from Hong Kong hospitals state that 32% of male and 15% of female COVID-19 patients required intensive care or died. On the other hand, the long-term fallout of coronavirus may be worse for women than for men due to social and psychosocial reasons. Regardless of sex- or gender-biased data obtained from WHO and those gathered from sometimes controversial scientific journals, some central points should be considered. Firstly, SARS-CoV-2 has a strong interaction with the human ACE2 receptor, which plays an essential role in cell entry together with transmembrane serine protease 2 (TMPRSS2); it is interesting to note that the *ACE2* gene lays on the X-chromosome, thus allowing females to be potentially heterozygous and differently assorted compared to men who are definitely hemizygous. Secondly, the higher *ACE2* expression rate in females, though controversial, might ascribe them the worst prognosis, in contrast with worldwide epidemiological data. Finally, several genes involved in inflammation are located on the X-chromosome, which also contains high number of immune-related genes responsible for innate and adaptive immune responses to infection. Other genes, out from the RAS-pathway, might directly or indirectly impact on the ACE1/ACE2 balance by influencing its main actors (e.g., *ABO* locus, *SRY*, *SOX3, ADAM17*). Unexpectedly, the higher levels of ACE2 or ACE1/ACE2 rebalancing might improve the outcome of COVID-19 in both sexes by reducing inflammation, thrombosis, and death. Moreover, X-heterozygous females might also activate a mosaic advantage and show more pronounced sex-related differences resulting in a sex dimorphism, further favoring them in counteracting the progression of the SARS-CoV-2 infection.

## 1. Introduction

SARS-CoV-2 belongs to the β-coronavirus family and the associated severe acute respiratory syndrome (SARS), like the previous SARS-CoV and Middle East respiratory syndrome (MERS-CoV), it may cause life-threatening diseases [1,2,3,4,5,6]. Data from past epidemiological studies match with emerging observations, indicating a large, sex-dependent gap in disease infection and outcomes for SARS-CoV-2 disease (COVID-19) as for previous SARS [7,8]. Moreover, people aged 60 and over suffer worse outcomes with mortality rates above 50% [9,10], and sex differences in incidence and higher fatality rates in males compared to females were also peculiar to earlier SARS. Conversely, infants and children experience mild symptoms and a better prognosis, without sex differences, and with a mildly elevated proinflammatory cytokines-storm in the early phase of the illness [11,12]. Accordingly, thrombosis due to coagulation unbalance or inherited or acquired thrombophilia is a rare event among children [13,14,15,16,17], and this could in part account for the micro-thromboembolic events or cardiac injury found in the elderly severest cases as well the rarer disseminated intravascular coagulation (DIC) [18,19,20], strongly substantiating heparin-based anticoagulant treatments in the selected severe cases [18,21,22]. On the other hand, the severity of COVID-19 worsens with advancing age for both sexes, possibly due to a dysregulation of the immune response, a difference in sex-hormones that has become less evident with age between sexes, or a considerable unbalancing in the coagulation/fibrinolytic system and endothelial dysfunction with aging [23,24,25,26]. Accordingly, in a mouse model, gonadectomy did not affect disease outcome in male mice, whilst ovariectomy or estrogen receptor antagonists caused increased mortality in females after SARS-CoV infection [7]. This observation also strongly agrees with the fact that females mount stronger innate and adaptive immune responses and are relatively more resistant to virus infections than males. The difference in the copy number of X-linked genes involved in the immune response and the presence of genes dedicated to disease susceptibility in males and females may account for any other possible sex advantage [27,28,29,30]. As concerns sex hormones, testosterone suppresses innate immune responses, whilst estrogens have immune-suppressive effect at higher levels and immune-stimulant activity at lower levels [27,31,32,33], with peculiar functions to contrast virus replication in selected tissues such as nasal epithelial cells in humans [34].

Less investigated, is whether differences in the efficiency of the SARS-CoV-2 cell-entry might account for at least part of the observed sex-gap. It has been reported that SARS-CoV-2 enters human cells using the SARS-CoV receptor ACE2 and a specific transmembrane serine protease 2 (TMPRSS2) for the spike (S) protein priming [35], an observation that makes the two biomolecules promising therapeutic targets, useful for establishing prevention programs [35,36,37]. It has been described how a modest ACE2 expression characterizes the upper human respiratory tract and that this should limit the receptivity of the virus [38,39]. It is controversial whether Metallopeptidase domain 17 (ADAM17, also known as TNFα-converting enzyme, TACE), involved in the ACE2 ectodomain shedding, by increasing the amount of soluble ACE2 might or might not counteract the virus entry and/or exclusively contribute to ACE1/ACE2 unbalancing, inflammation and thrombosis [40]. Furthermore, estrogens increase ADAM17 and ADAM10 expression levels, two putative shedders also responsible for many ectodomain cleavages in atherosclerosis [41], suggesting their protective role against cardiovascular events in females, a mechanism potentially accounting for the observed COVID-19 sex-disparity.

The efficiency in the ACE2-receptor/S-protein recognition and interaction is a key determinant for the success of viral infection and receptivity [35]. Recently, the S-protein/ACE2 interface has been elucidated at the atomic level and several crucial amino acid residues are recognized to correctly achieve the homo-dimerization process [42]. Therefore, proper ACE2 functionality is essential for both virus cell entry and local pulmonary homeostasis, and although it has been previously described that polymorphisms in the *ACE2* gene do not affect the outcome of SARS [43], females might have a higher degree of heterodimer assembling than males, which in turn might show different affinity for the SARS-CoV-2 spike receptor.

Similarly, single nucleotide polymorphisms (SNPs) within the *TMPRSS2* gene (21q22.3) can also have a greater role in the general population (rs2070788, rs7364083, rs9974589) and in a sex-oriented perspective (rs8134378) hypothesizing that higher expression in males might favor virus membrane fusion, *TMPRSS2* being an androgen responsive gene [44,45] in line with previous GWAS on A(H1N1) and A(H7N9) influenza [46]. Conversely, estrogen fall in postmenopausal females in turn affects *TMPRSS2* expression, the gene also being responsive to estrogens [47].

**Figure 1:**
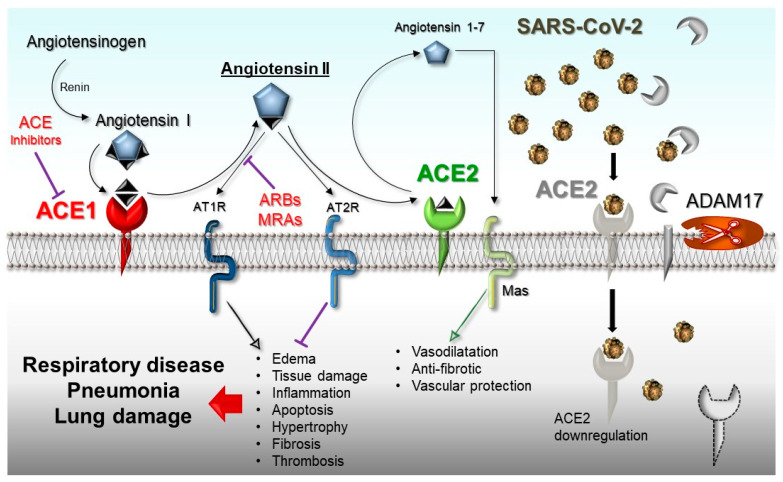
Schematic representation of the renin–angiotensin system (RAS)-pathway in which ACE1/Ang-II/AT1R-axis and ACE2/Ang 1-7/Mas-axis are shown. On the right of the panel, the SARS-CoV-2-mediated suppression of the ACE2 receptor and the cleavage activity of ADAM17 on ACE2 are shown. ADAM17: Metallopeptidase domain 17; ARBs: Angiotensin receptor blockers; MRAs: mineralocorticoid receptor antagonists.

Finally, ACE1 and ACE2 cooperate in the renin–angiotensin system (RAS) to balance the local vasoconstrictor/proliferative (ACE1/Ang-II/AT1-axis) and vasodilator/antiproliferative (ACE2/Ang1-7/MAS-axis) actions. This results in the protection of organs and blood vessels by anticoagulant, anti-inflammatory, anti-proliferation, anti-fibrosis, anti-alveolar epithelial cell apoptosis, and anti-oxidative stress activities antagonizing the Ang-II effects (Figure 1).

Therefore, the coexistence of inherited predispositions or common gene polymorphisms in the *ACE1* and *ACE2* genes that affect their mutual expression levels might lead to increased capillary permeability, coagulation, fibrosis, and apoptosis in the alveolar cells, accelerating lung damage and pulmonary shut-down triggered/worsened by the SARS-CoV-2 infection.

Most likely, it is plausible that a combination of the mechanisms described above might influence the multistep pathogenesis and the age/sex-gaps of such a complex infection and progression, considering also that *ACE2* (locus Xp22.2) and Ang-II receptor type 2 gene (*AGTR2*, alias *AT2*, locus Xq23) are both located on the X-chromosome. Essentially, X-linked heterozygous alleles could activate in females a mosaic advantage and a greater sexual dimorphism that might counteract viral infection, local inflammation due to cytokine storms and severe outcomes.

In the present work, we will address the hypotheses suggested above and reveal the main underlining genetic mechanisms with the hope that a sex-oriented approach will add new insights for a better understanding of COVID-19 also in the general population.

## 2. ACE1/ACE2 Pathway and Acute Respiratory Distress Syndrome (ARDS)

The renin–angiotensin system (RAS) acts as a homeostatic regulator of the vascular function, including blood pressure and volume control, starting with renin (REN) mediating the transformation of angiotensinogen (AGT) to angiotensin I (Ang-I). Indirectly, RAS is also responsible for local tissue homeostasis by anti-inflammatory, anti-coagulant, anti-proliferation, anti-fibrosis, anti-apoptosis of epithelial cells, and anti-oxidative stress activities, also controlling the local trophic responses to a range of stimuli, viruses included [48,49].

The angiotensin I-converting enzyme (ACE1) and the more recently discovered homologue ACE2 [50,51], are two antagonist enzymes of the RAS pathway that act and counterbalance each other [52]. The main role of ACE1 is the conversion of angiotensin I to angiotensin II (Ang-I > Ang-II), the latter being a powerful peptide causing complex processes such as vasoconstriction, inflammation, fibrosis and proliferation via the AT1-receptor. Conversely, ACE2 firstly converts Ang-I to Ang 1–9, that is then converted by ACE1 in the vasodilator peptide Ang 1–7. Moreover, ACE2 directly converts Ang-II to Ang 1–7 and this latter by acting on MAS-receptor exerts organ protection, antagonizing the biological effects of Ang-II [50,51,52]. Moreover, a high ACE2/ACE1 ratio protects against endothelial dysfunctions and vascular pathologies, exogenous ACE2 activation promotes antithrombotic activity, and the known antithrombotic properties of captopril (ACE inhibitor) and losartan (AT1-receptor blocker) are attenuated by a selective Ang 1–7 receptor antagonist [53,54].

ACE2 is a key molecule for the tuning of the RAS pathway under both healthy and pathological conditions. It has been recently demonstrated that SARS-CoV-2 uses the same receptor (ACE2) as SARS-CoV to enter cells in combination with the action of the serine protease TMPRSS2 for S-protein priming [35]. *ACE2* is expressed in several tissues, including endothelium, lung, heart, intestine, and kidney and, as recently demonstrated, on the epithelial cells of oral mucosa and the tongue [36], sharing both tissue expression sites and high sequence identity with the homologue *ACE1* [50,51]. Anomalous tuning of the ACE1/ACE2 pathway contributes to the development of several complex pathological conditions such as hypertension, atherosclerosis, thrombosis, heart or kidney failure, and severe acute respiratory distress [48]. In the lung, *ACE1* is highly expressed, and *ACE2* is mainly clustered in type-II alveolar cells [36,55,56,57]. During acute respiratory distress syndrome (ARDS), a local RAS unbalance cannot maintain appropriate oxygenation, thus inducing pulmonary edema, inflammation, and hyper-proliferation, establishing in turn severe pulmonary shutdown [48,52,58].

The observations from the previous SARS epidemic (2003–2009) that some coronaviruses use heparan sulfate as a receptor entry by acquiring heparan sulfate-binding sites, and that the heparin molecule acts as competitor preventing the binding of the spike protein to the host cell, inhibiting infection rate and mortality, are a valuable rationale to start heparin treatment in selected COVID-19 patients [59,60,61,62]. This approach might have a double result, to reduce virus entry and avert thrombotic complications or organ dysfunctions [18,21,22].

**Figure 2:**
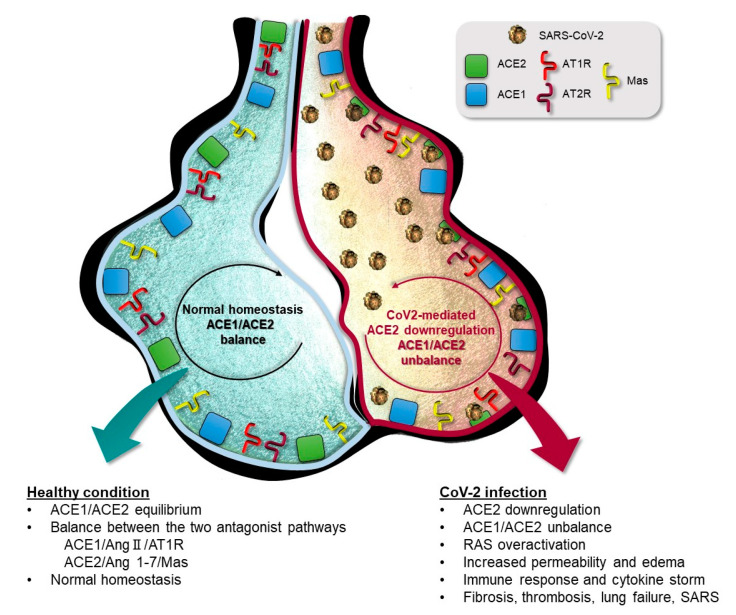
Schematic representation of lung alveolar in presence of normal homeostasis (left) characterized by a balanced ACE1/ACE2 pathway, and during SARS-CoV-2 infection condition (right) in which SARS-CoV-2 mediated suppression of ACE2 receptor causes ACE1/ACE2 unbalance responsible for RAS over-activation and pulmonary shut-down.

Interestingly, in ARDS animal models, *ACE2* knockdown mice experience more severe symptoms [58]. *ACE2* gene deletion causes progressive cardiac fibrosis [63], whereas *ACE2* deficient mice result in renal injury and glomerulosclerosis [64,65]. These alterations are reversed or ameliorated by treatment with ACE-inhibitors or Ang-receptors blocker (ARBs) [37,63,66,67], whether or not they are combined with infusion of a soluble form of recombinant human ACE2 (rhACE2) [68,69,70]. Intuitively, this strategy could also be a possible treatment for COVID-19 patients with a double effect: excessive soluble rhACE2 could competitively bind and neutralize the SARS-CoV-2 virus, and rescue the cellular ACE2 activity counteracting unescapably unrestrained ACE1 activity to contrast lung injury. In summary, the ACE1/ACE2 balance is crucial in contrasting organ dysfunction, so a direct or indirect increase in ACE2 expression together with a modulation of ACE1 activity may be helpful to avoid pulmonary disease progression (Figure 2) [37,58,71,72,73,74]. Accordingly, several international societies recommend not to stop treatments with RAS pathway antagonists in cardiovascular disease patients [40], and a recent editorial comments on several publications discussing the positive effects of RAS inhibitors during COVID-19 [75].

Finally, other genes directly or indirectly regulate the RAS pathway (Table 1). Firstly, *ADAM17* by promoting the detaching of ACE2 cell receptor might contribute by downregulating the ACE2/Ang1-7/Mas axis, and in a sex-oriented perspective, *SRY* (Y-chromosome) and *SOX3* (X-chromosome) both by upregulating *AGT*, and downregulating *ACE2*, *AT2*, and *MAS*. Conversely, *SRY* upregulates, whilst *SOX3* downregulates, the *REN* promoter, thus being a potentially detrimental step in limiting the global rate of the RAS system that is particularly frail in males [76,77].

### ACE1 and ACE2 Genes

*The ACE1* gene maps on chromosome 17q23.3, is 21.32 kb-long, and comprises 26 exons (Genbank, NT 010783). NCBI records (https://www.ncbi.nlm.nih.gov/SNP/snp_ref.cgi?locusId=1636) include hundreds of intragenic gene variants: most of them are single nucleotide polymorphisms (SNPs), a minor part of which are located in the coding region, and few of them are missense mutations. Interestingly, the gene encodes two isoforms of ACE1 with two different promoters and alternative splicing, and the structure of the *ACE1* gene may be the result of a duplication of an ancestral gene [78].

Among the several gene variants, the more investigated is an insertion/deletion (I/D) defined by NCBI [79] as a sequence of 287-bp I/D in the *Alu*-sequence of intron 16 in the *ACE1* gene and is represented by four individual SNPs (rs4646994, rs1799752, rs4340 and rs13447447). It is generally associated with the expression level of ACE1 [80], with the D/D genotype having the highest serum/tissue ACE1 levels, the I/D genotype showing intermediate levels, and the I/I genotype the lowest levels [81]. Interestingly, the Online Mendelian Inheritance in Man (OMIM), a catalogue of human genes and genetic disorders particularly focused on gene–phenotype relationships, reports that this gene may be associated with SARS progression, assuming that the D-allele can be considered a genetic predisposition affecting the progression from pneumonia to SARS, as reported in a study with Vietnamese SARS patients [82]. Other studies have confirmed these outstanding data [82,83,84], although current conclusions are still controversial [43,85,86].

Although about 60% of the ACE1 levels seem to be determined by the *ACE1* I/D polymorphism, it is extremely interesting that there is a significant sex-difference in serum ACE1 activity/level, which is lower among females in both healthy and pathological conditions [81,87,88,89]. This evidence, together with the observation that the *ACE1* I-allele seems to be overrepresented among females, and that the D-allele (associated with a high level of ACE1) seems more prone to express even higher levels among males, suggests a higher chance to have ACE1/ACE2 imbalance among males during ACE2 receptor suppression, as in the presence of SARS-CoV-2 infection [35,37].

Interestingly, a quantitative variation in ACE1 levels has been demonstrated to be modulated also by the *ABO*-blood group locus (9q34.2). Moreover, selected *ABO* polymorphisms (rs495828, gene promoter, and rs8176746, exon 7) influence ACE inhibitors treatment response [90,91,92], and might contribute in reducing SARS-CoVs’ transmission, in terms of number of infected individuals and epidemic rate reduction. This ascribed to O-blood group a lower risk of infection, hypothesizing that natural anti-A and anti-B antibodies can contribute in protecting against viral diseases at the population level [93].

The *ACE2* gene maps on chromosome Xp22.22, is 41.04 kb long, and contains 18 or 19 exons in two isoforms (Genbank, NT 011757), which differ in the presence of an extra exon in the longer isoform at the 5′-end [51]. The ACE2 and ACE1 catalytic domains share 42% homology in their amino acid sequence and have a similar exon/intron organization, indicating a common ancestor. Among the several gene variants [94], particular attention has been paid to the transition G8790A (rs2285666) in intron 3 of the *ACE2* gene, previously investigated in association studies with hypertension, although some issues remain almost inconclusive [95,96]. Unlike autosomal genes (i.e., *ACE1*), X-linked genes (i.e., *ACE2*) cannot show in males any advantageous heterozygous condition in case of mutations or polymorphic at-risk conditions. Accordingly, in the presence of a lower activity of the *ACE2* gene, as for the one associated with the 8790 G-allele [97,98], male-carriers are certainly hemizygotes unable to compensate with the 8790 A-allele counterpart. *ACE2* G8790A is located at the beginning of the intron 3, theoretically affecting gene expression with alternative splicing mechanisms [99,100], also having a strong linkage disequilibrium with other SNPs (rs1978124 intron 1 and rs714205 intron 16) in the *ACE2* gene [101,102].

An interesting recent paper investigated the *ACE1* I/D variant in combination with the *ACE2* G8790A transition in hypertensive population. The authors hypothesized that patients characterized by higher ACE1 activity (i.e., D/D-genotype) in conjunction with reduced ACE2 activity (i.e., GG-females or hemizygous G-males) could account for increased susceptibility to hypertension mainly in association with classical cardiovascular risk factors such as old age, dyslipidemia, and diabetes [97].

**Figure 3:**
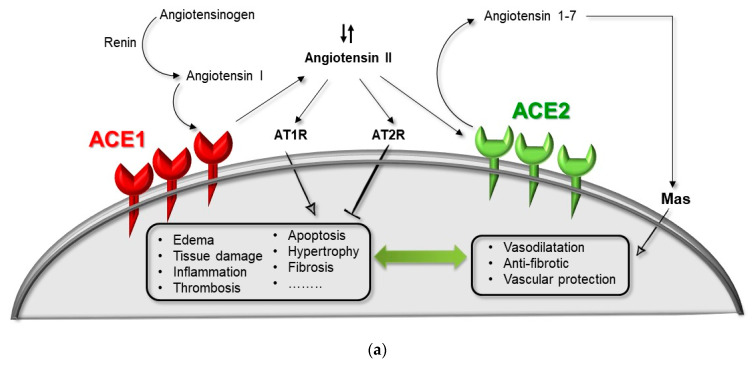

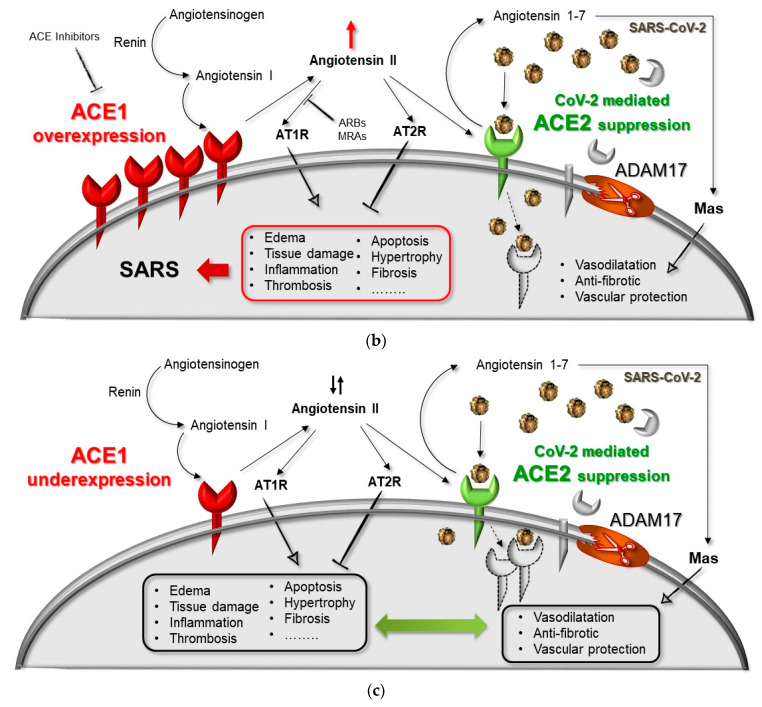
Hypothesized mechanism of a genetic–environmental interaction between ACE1/ACE2 genes and SARS-CoV-2 infection. (**a**) Normal-health condition with a balanced RAS, normal Ang-II levels (Ang-II ↓↑), in the absence of virus infection. (**b**) Over-expression of ACE1 receptor as in the presence of the *ACE1* DD-genotype (i.e., rs4646994, rs1799752, rs4340, rs13447447) in combination with ACE2 downregulation due to SARS-CoV-2 infection and/or in the presence of the *ACE2* 8790 G-allele (i.e., rs2285666), resulting in ACE1/ACE2 unbalancing, RAS over-activation (Ang-II ↑) and lung shut-down. (**c**) Under-expression of ACE1 receptor as in the presence of the *ACE1* II-genotype (i.e., rs4646994, rs1799752, rs4340, rs13447447) in combination with ACE2 downregulation due to SARS-CoV-2 infection counteracted by the *ACE2* 8790 A-allele (i.e., rs2285666), resulting in ACE1/ACE2 re-balancing, no RAS over-activation, re-balanced Ang-II levels (Ang-II ↓↑), and no lung shut-down. ADAM17: metallopeptidase domain 17; ARBs: angiotensin receptor blockers; MRAs: mineralocorticoid receptor antagonists.

Noticeably, it emerges that an overly activated RAS, exacerbated by genetic predispositions affecting the ACE1/ACE2 balance and in combination with advanced age and classic acquired cardiovascular risk conditions, might lead to systemic disorders and/or severe local disturbances of the normal tissue homeostasis. Overall, this condition can establish a complex multistep mechanism (Figure 3) leading to organ dysfunction such as in SARS-CoVs where the ACE1/ACE2 equilibrium is destroyed and incidentally worsened by the suppression of the ACE2 receptor due to SARS-CoV-2 binding, and/or ADAM17 activity, and/or by the combination of specific *ACE1*/*ACE2* at risk haplotypes [35,37,48,71,103].

Our hypothesis should be demonstrated by dedicated clinical epidemiological studies in genetically selected patients, though it is to be taken into account that the above described *ACE1* and *ACE2* gene variants do not completely lessen the enzyme levels. Rather, they are responsible for a medium–mild reduction with enzyme levels within the low–normal range of the general population. Nonetheless, in critical conditions and co-morbidities (e.g., SARS-CoV-2 infection), selected genetic variants might favor RAS-rebalancing. Moreover, among COVID-19 patients, those taking RAS-inhibitors may take advantage of the treatment considered an adjuvant drug for RAS rebalancing [40,75].

Finally, the surface of SARS-CoV-2 interacts specifically with ACE2 through its receptor binding domain (RBD) of the S-protein, which is critical to the success of the viral infection. The affinity between ACE2 and the RBD of the SARS-CoV-2 is 10–20 times higher than that of previous SARS-CoVs, which also explain its higher aggressive performance [104,105]. Moreover, the ACE2 peptidase domain (PD), normally deputed to Ang-I to Ang 1–9 cleavage, also makes available a direct binding site for SARS-CoV-2 S-proteins. In addition to the PD domain, a neck domain is also crucial for the ACE2 dimerization process and stability. Extensive polar interactions have been recognized in the neck domain of ACE2, and the complex network of polar interactions between amino acid residues ensures a stable and functional dimer assembly. Several gene polymorphisms have been listed among those amino acid residues suggested to be crucial for dimer stability in the PD and neck domains of the *ACE2* gene [106,107]. Theoretically, this might interfere with the ACE2-SARS-CoV-2 interaction, especially in females who have two different *ACE2* genes within the two different X-chromosomes, a condition that may give rise to a greater structural variability in females, who potentially assemble “hetero-dimers”, particularly in the presence of a skewed X-chromosome inactivation (XCI), in comparison to males who necessarily assemble “homo-dimers”. Obviously, XCI is not the same in every female, being potentially advantageous or not in terms of virus cell entry. Moreover, a difference in the open or closed conformation of the ACE2 receptor together with the glycosylation rate of some amino acid residues in the PD domain could affect ACE2-SARS-CoV-2 interaction [105,108], as demonstrated by chloroquine treatment of SARS-CoV patients, in which chloroquine inhibited virus infection by interfering with the terminal glycosylation of ACE2 receptor [109]. Accordingly, we have previous experience of dimeric/tetrameric molecules in which selected SNPs seemed to have higher detrimental effects on the molecule structure and activation when combined heterozygous haplotypes were co-inherited in the same carrier rather than in homozygous polymorphic individuals [110,111,112,113,114,115,116].

## 3. Immune Processes: An X-Related View

Sex-disparities have been often observed in response to communicable diseases [107,117]. In viral infections, sex differences in term of intensity, prevalence, severity, and mortality have been reported depending on the etiological agent, as reviewed in [118]. The general tendency in females is, however, to show a greater humoral and cell-mediated immune response to infection [117,118,119]. Consequently, females clear infections more quickly and more effectively than males [118,120,121]. Sex hormones can be partly responsible for this phenomenon: by binding to specific receptors on immune cells, they can trigger their activity (estrogen) or suppress it (testosterone) [119,122,123]. However, sex differences may also be due to an imbalanced expression of genes on the X- and Y-chromosomes, since immune-related X-linked genes appear to be more activated in female immune cells.

The sex chromosomes X and Y, which differentiated from ordinary autosomes about 180 million years ago, show great levels of dissimilarities, except in their pseudo-autosomal regions (PAR1 and PAR2) located at the end of both chromosomes [123]. Having two X-chromosomes, females have two different cell types in all their organs with one of the two X-chromosomes being inactivated (Xi, while the activate form is Xa), a condition that was first described by Mary F. Lyon in mice [124]. The XCI is due to epigenetic processes that randomly select and permanently silence one of the two X-chromosomes of a female individual. The process occurs at an early phase of embryonic development and is maintained during adulthood. XCI is a strategy that aims to balance the X-linked transcriptional dosage between female XX and male XY cells. In general, 50% of female cells and all cells derived from them have the maternal X-chromosome inactivated, the other 50% have the paternal X-chromosome inactivated. The knowledge of the complex mechanism of XCI and its regulation is summarized in [125]. XCI results in a condition of “cell mosaicism” in a female individual, where a balanced expression of both parental X-linked genes is expected [126]. This condition may provide females with greater plasticity and adaptability in the response to infectious diseases than males. When it comes to gene expression, females may compensate adverse X-linked mutations by using cells that carry the wild allele on the other X-chromosome [124]. However, it is clear that XCI is incomplete in humans [127] and that the balance may be disrupted both by the process of skewed XCI and by genes escaping silencing [123].

Skewed X-chromosome inactivation appears when one X-chromosome is favored over the other for XCI. This may result in 75–90% of cells with the same parental X-chromosome silenced. This predominance of one Xi also causes unbalance of the allelic dosage and of X-linked transcription. This loss of mosaicism may lead to a reduced plasticity of the immune system and a loss of immunological memory [126]. There are several mechanisms underlying skewed XCI which appear to be partly age-dependent, showing an increment in older women, at least in the blood, which is also associated with smoking [126].

With regard to the mechanism of escaping silencing, it seems that about 15% of X-linked genes can escape inactivation, so that they are expressed by both X-chromosomes in female cells, whereas a further 10% vary among individuals for their silencing behavior. The level of expression is, however, always lower in the escaped gene [128]. XCI escape signatures vary between individuals, even during growth and aging, as well as between cells in a tissue [128].

In humans, naïve mature B and T cells were demonstrated to lack XCI on some genes (H3K27me3, H2A-ubiquitin, H4K20me and macroH2A), which are again partially silenced (H3K27me3 and H2A-ubiquitin) when lymphocytes are activated [117]. Other immune cells have shown evidence of different XCI maintenance in females, as summarized in [117]. Essentially, the results of different studies now support the theory that sexual dimorphism of immune cell is triggered by XCI escape mechanisms [117]. In particular, the distal end of Xp and PAR regions, which contain the highest number of immune-associated genes on the X-chromosome, have a higher chance of escaping XCI [123].

Accordingly, in the *ACE2* gene maps of the Xp-region and in a study carried out without distinguishing between male and female cells [39] it was demonstrated that *ACE2* is abundantly present in human lung epithelia and small intestine enterocytes, while another study [36] has shown that the expression of the ACE2 receptor is higher on oral mucosa and tongue epithelial cells. A systematic survey of XCI that integrated transcriptomes with genomic data [127] identified *ACE2* as a tissue-specific escape gene that showed moderate male-biased expression in lungs, higher male-biased expression in the small intestine, and weak male-biased expression in Epstein–Barr virus (EBV)-transformed lymphocytes. This finding may be suggestive of lower *ACE2* expression in females due to the combination of the two X-linked genes compared to the expression arising from the X-linked and a Y homologue in males [127]. Nevertheless, in another recent study [129], no sex-mediated differences in expression were found between the sexes. Alternatively, the predominant male-biased expression of *ACE2* might be explained by increased *ACE2* activity in males partially driven by sex hormones [127], as it has recently been demonstrated in mice kidneys [130].

Finally, strong results have recently been found in favor of an exceptionally high basal level of ACE2 in Asian females than in other ethnic groups and an age-dependent decrease more significant in men than women [131]. Briefly, this study shows an apparent negative correlation between ACE2 quantitative expression and infection susceptibility and severity at population level [131]. Nevertheless, the tissues they have tested are not those of the upper respiratory tract, with the exception of the lung (and blood vessels) which only show a moderately higher ACE2 expression in East Asian females. The authors’ conclusion that Asian women are more protected against CoVs than men is based on the hypothesis that ACE2 repression induced by SARS-CoV2 might be counteracted by high basal levels of ACE2 induced by higher levels of sex hormones (which decrease with age) and reduced by systemic inflammation [131]. This fascinating theory, which would support the hypothesis of ACE1/ACE2 unbalance on ARDS onset in COVID-19 patients, needs to be confirmed in the future when more data will be available.

## 4. Inflammatory Processes: An X-Related View

Sexual dimorphism in terms of risk, susceptibility, and prognosis has been reported for several inflammatory pathological conditions, including respiratory diseases in which hormonal profile, genetic, and epigenetic factors may play a role [8,27,132]. In this scenario, it is relevant that the response to vaccination seems to be improved in females who therefore show a lower risk/vulnerability to several pathogens, while males are more exposed and at higher risk [133]. Furthermore, evidence suggests that males tend to experience the worst prognosis in acute inflammatory settings (e.g., sepsis) [134,135], while there is a generalized inversion of prognosis between men and women in chronic inflammatory conditions (e.g., asthma) [136,137]. In relation to SARS-CoV2, the accumulation of reports and epidemiological data indeed confirms that, in agreement with other respiratory inflammatory diseases and consistent with previous MERS-CoV and SARS-CoV infections, the new coronavirus preferentially affects males than females that show a better prognosis [138,139,140].

The onset of inflammatory processes is a key pathological feature of SARS-CoV-2 infection. The massive release of inflammatory cytokines and chemokines in COVID-19 patients was first reported by Huang and colleagues, showing in particular higher levels of IL2, IL7, IL10, GSCF, IP10, MCP1, MIP1A, and TNFα in the peripheral blood of intensive care unit (ICU) patients compared to non-ICU patients [141]. The impact of the “inflammatory-wave” in COVID-19 suggests that the cytokine storm might be strongly associated with the severity of the disease [141]. The role of the cytokine storm in SARS-CoV-2 infection is attracting great attention as a crucial phase to investigate in order to clarify the disease’s pathogenic process (e.g., to elucidate the role of Th1 and Th2 responses) and to identify new therapeutic targets (see the published results and the ongoing clinical trials with Tocilizumab, a monoclonal antibody targeting the IL-6 pathway) [141,142,143]. Sex hormones have been suggested as potential mediators of the reported sexual dimorphism by virtue of their ability to modulate innate and adaptive immunity, as previously reported [144] and as described above. However, since sex dimorphism observed in several inflammatory diseases, including respiratory pathologies, has also been confirmed in studies dealing with pre-pubertal cohorts [145,146,147], there is growing consensus on the need to consider additional factors/variables that may occur. Similarly, there is growing evidence suggesting that the X-chromosome and X-linked genes are the main determinants of the reported sex dimorphism in disease susceptibility and prognosis [8,148]. The X-chromosome carries about 1,200 genes [149] including cytokines/cytokines receptors, toll-like receptor (TLR)-mediated signaling pathway genes, NF-kB and MAPK signaling genes, genes involved in apoptosis, genes involved in redox balance, and other immune-modulators such as CD40 ligand and FOXP3, as recently reviewed [150].

With regard to the role of the number of X-chromosomes on inflammation and release of inflammatory cytokines, in a recent work Lefèvre and colleagues addressed how the number of X-chromosomes can affect the secretion of inflammatory cytokines after the activation of the TLR signaling pathways [151]. The authors demonstrated that cytokine production in response to different TLR ligands was improved in males, showing higher inflammatory response than in females and subjects with Klinefelter syndrome who carry two X-chromosomes, but are characterized by a hormonal profile more similar to that of males [151]. The presence of two X-chromosomes carrying genetic variants of X-linked inflammatory genes/receptors undergoing random inactivation may therefore represent an advantage in the acute phase of inflammatory diseases [151,152].

Finally, X-linked genes coding for inflammatory mediators/receptors may also escape XCI leading to bi-allelic expression [153], with strong implications in sex-related differences of inflammatory responses [154]. Interestingly, the X-linked *ACE2* gene, in transgender males treated with estrogen therapy and androgen antagonists shows significantly higher expression and increased number of cells expressing *ACE2* among testicle cells [131]. Overall, if inflammation is needed to control the invasion/elimination of pathogens, the onset of exacerbated inflammatory responses may be harmful and lead to tissues/organs damage. In this scenario, females result as better “armed” thanks to the presence of a cellular mosaicism that may be more than useful to modulate/balance inflammation [150].

The lessons learned from different respiratory diseases of viral and non-viral origin, and preclinical research suggest that the number of active X-chromosomes can make a difference in shaping the type, dimension, and lasting of inflammatory responses that, in synergy with sex hormones, may account for the low risk and better prognosis of SARS-CoV-2 infection in females, though a multidisciplinary approach for such complex condition is mandatory.

## 5. Habits, Gender and Environmental Related Risks

According to international disaggregated data, men are faring worse than women in COVID-19 pandemic [155]. On the other hand, the long-term coronavirus fallout may be worse for women than for men due to social, psychosocial, and occupational reasons as well as environmental factors and differences in lifestyle. Biological and behavioral causes directly or indirectly play a role in modifying women’s risk such as different comorbidity rates (e.g., hypertension and cardiovascular diseases), smoking and drinking habits, personal hygiene, the number of females among nurses and caregivers both in hospitals and within families, and of course pregnancy and motherhood, just to cite the most relevant.

Within the several habit-related factors, smoking deserves particular consideration. Smoking is a risk for many respiratory diseases and undoubtedly also for COVID-19 [155,156]. The European Respiratory Society [157] reported results of different studies concluding that smokers are 14 times more likely to die from COVID-19 [158,159], but only a few studies definitely advise to stop smoking [160] and, to our knowledge, no country has taken strong prevention measures. As regards the individual susceptibility of smokers toward respiratory diseases, such as influenza, chronic obstructive pulmonary disease (COPD), tuberculosis (TB), or other lung pathological conditions, there are no doubts about a direct association [161,162,163,164]. Accordingly, the European Centre for Disease Prevention and Control (ECDC) suggested that smoking may contribute to increasing the number of severe COVID-19 cases [165]. Conversely, it has been speculated that smokers seem less likely to develop COVID-19, by the hypothesis that smoke exposure might modulate the immune response and the levels of inflammation markers. By attenuating the physiological defense of the immune and inflammatory system, smoking might paradoxically mount a less aggressive cytokine storm [166]. Accordingly, a “nicotinic hypothesis for COVID-19” has been speculated, suggesting the use of nicotine-patches on coronavirus patients [167]. Overall, no evidence exists that smoking protects COVID-19 patients from developing severe symptoms and recent metanalyses indeed list smoking habits as risk factors [168,169,170]. Of note, it has been reported that the ACE2 receptor is upregulated in the lungs of smokers or COPD patients, including small airway epithelium, brush borders, type-II alveolar pneumocytes and alveolar macrophages [171,172]. The expression was more evident in patients with COPD compared to never-smokers, suggesting that smoking upregulates ACE2 expression and COPD further exacerbated it, hypothesizing in them an amplified susceptibility for COVID-19.

Environmental and health issues may result associated with COVID-19 and some of them deserve to be mentioned [173]. First of all, air pollution and poor air quality has been suggested to lead to an increased COVID-19 infection rate probability due to a direct effect of air pollution on humans [174]. Moreover, other studies on flu-like viruses highlighted the potential role of pollen in the atmosphere, that by increasing general immune responses might affect viral spread, speculating that it might also decrease during warm seasons [175]. Hospitals are crucial in COVID-19 pandemic containment and controlling, and for dedicated disinfection techniques for infrastructures personnel and medical equipment are key issues [176] to avoid front-line healthcare worker infection risk and increased psychological burden, particularly for medical doctors and nurses among which women account for the largest number worldwide [177]. Overall, protective measures for people at such a high risk as hospital workers is of primary importance. Efficient personal and protective equipment reduces mortality, and we need to plan strategies to ensure protection also against infections rising from environmental sources. Therefore, any useful tool/instrument/product/action should carry a global benefit.

Finally, considering individual genetic susceptibility or environmental influences on virus infection and/or SARS progression, a wide and heterogeneous geographical distribution of absolute numbers and percentages of infected cases is now emerging. Different clinical phenotypes and symptoms characterize not only different populations and countries, but also close geographic regions in which opposite situations may coexist. As a paradigmatic example, according to data from “*Dipartimento Protezione Civile COVID-19 Italia*” [178], our region, Emilia Romagna, is the second area of Italy for absolute number of SARS-CoV-2 infections (26,487 confirmed cases/4,452,638 habitants; male/female ratio 48.6/51.4). It accounts for an overall infection rate of about 0.59%, characterized by 3,366 deaths (14.2% death rate), considering 217,039 performed tests and 1899 total recovered patients (7 May 2020, 7:00 PM). Among the towns belonging to the same region, Ferrara shows the lowest percentages (960 confirmed cases/346,975 habitants; male/female ratio 48.1/51.9) accounting for an overall infection rate of about 0.27%, compared with the town of Piacenza (less than 200 Km far from Ferrara) which has the highest rate (4300 confirmed cases/286,781 habitants; male/female ratio 48.9/51.1) accounting for an overall infection ratio of about 1.5% (Ferrara vs. Piacenza; *p*< 0.00001; Ferrara vs. rest of the region; *p* < 0.00001). Obviously, other variables also need to be analyzed, first the local containment actions, the number of tests performed and then the geographic locations. In this regard, Piacenza is very close to the most critical hot-spot of the infection in Italy [region Lombardia (0.83%); Lodi (1.43%), Codogno area (red zone)] and other additional parameters such as population density may account for local differences in terms of viral spreading (Ferrara: 325 habitants/Km^2^ vs. Piacenza 880 habitants/Km^2^). Genetic selection pressure by previous epidemics, such as the long-lasting malaria epidemics in the Po river area where the city of Ferrara is located, cannot be excluded. This is in line with some publications reporting that endemic malaria seems to protect from COVID-19 outbreak and that genetic variations associated with malaria (e.g., *ACE2* and *ABO* genes) may play protective roles [179,180,181].

In summary, individual susceptibility, mainly driven by gene-environment interactions, has a great role in determining immunity, survival, and treatment responses in different populations.

## 6. Conclusions

The COVID-19 pandemic by coronavirus SARS-CoV-2, is a severe, complex, and multifactorial disease in which human genetics, due to inherited predispositions, could play a role together with pre-existing comorbidities and acquired risk conditions. Unmodifiable factors, such as age and sex, together with modifiable classical cardiovascular risk factors and comorbidities play crucial roles in tuning the fate of SARS-CoV-2 infection, worsening prognosis, and mortality rate. In turn, the mechanism of SARS-CoV-2 infection, due to ACE2 receptor entry, might be altered by individual genetic or developed susceptibility. An overly activated RAS-system, mainly due to local ACE1/ACE2 unbalancing, might be crucial in determining specific vulnerability/receptivity and in giving indicators of the local balanced inflammation, blood coagulation, and fibrinolysis. These evidences support the use of appropriate anticoagulant and anti-inflammatory therapies properly monitored by specific laboratory markers (as fibrin D-Dimer) or with a more refined approach by the dynamic assessing of residual FXIIIA levels, as recently suggested during any acute thrombotic event [182,183]. The unexpected reported higher expression of *ACE2* in females, the inverse age-dependent *ACE2* expression significantly reduced by the presence of diabetes, and the strong repression of *ACE2* by inflammatory cytokines, confirm an inverse correlation between *ACE2* levels and SARS-CoV-2 prognosis. Summarizing, this is in contrast with the assumption that “*high ACE2 is a culprit in COVID-19 outcome, and on the contrary supports a protective role of high ACE2 expression against SARS-CoV2 fatality*” [131] also strengthened by the ACE2 intrinsic anticoagulant properties. Interestingly, SARS-CoV-2 mediated repression of ACE2 is counteracted by sex hormone inducible mechanisms (though unfortunately sex-hormones decrease with age) and exacerbated by systemic inflammation (which unfortunately increases with age and chronic diseases) [131].

Part of the present findings were already known from previous SARS epidemics, but the pandemic proportions of COVID-19 have led researchers and clinicians to fight together more than during previous SARS-CoVs infections. Accordingly, some aspects such as the sex gap in favor of the females, particularly for prognosis and survival, have been greatly deepened and investigated through epidemiological studies. Different sex and age groups have strongly different susceptibilities to infection and mortality, considering male-sex, old age, and comorbidities as the most affecting variables. In addition, genetic predispositions or inherited protective mechanisms have been speculated to explain some controversial data. Considering individual genetic susceptibility to virus infection and progression, heterogeneous geographical distributions are now emerging not only among different populations and countries, but also between close geographic regions.

In conclusion, while waiting for an effective and safe vaccine the main task is to understand the individual virus susceptibility/receptivity together with the repurposing of already available pharmacological compounds, such as selected anti-inflammatory, anti-malaria, and anticoagulant drugs as well as reconsidering RAS-antagonists in selected patients to achieve efficient and personalized targeted therapies.

## Figures and Tables

**Table 1:** Main genes directly or indirectly involved in the Renin Angiotensin System homeostasis.

Gene	HGNC ID	Name	Locus
*AGT*	333	Angiotensinogen	1q42.2
*REN*	9958	Renin	1q32.1
*ACE1*	2707	Angiotensin I converting enzyme	17q23.3
*ACE2*	13557	Angiotensin I converting enzyme 2	Xp22.2
*AGTR1 (AT1)*	336	Angiotensin II receptor type 1	3q24
*AGTR2 (AT2)*	338	Angiotensin II receptor type 2	Xq23
*MAS1*	6899	MAS1 proto-oncogene	6q25.3
*ABO*	79	α 1-3-N-acetylgalactosaminyltransferase α 1-3-galactosyltransferase	9q34.2
*ADAM17*	195	Metallopeptidase domain 17 TNFα-converting enzyme (TACE)	2p25.1
*SRY*	11311	Sex determining region Y	Yp11.2
*SOX3*	11199	SRY-box transcription factor 3	Xq27.1

HGNC: Human Gene Nomenclature Committee of the Human Genome Organisation (HUGO).
